# CD4-Transgenic Zebrafish Reveal Tissue-Resident Th2- and Regulatory T Cell–like Populations and Diverse Mononuclear Phagocytes

**DOI:** 10.4049/jimmunol.1600959

**Published:** 2016-09-30

**Authors:** Christopher T. Dee, Raghavendar T. Nagaraju, Emmanouil I. Athanasiadis, Caroline Gray, Laura Fernandez del Ama, Simon A. Johnston, Christopher J. Secombes, Ana Cvejic, Adam F. L. Hurlstone

**Affiliations:** * Faculty of Life Sciences, The University of Manchester, Manchester M13 9PT, United Kingdom;; † Wellcome Trust Sanger Institute, Wellcome Trust Genome Campus, Hinxton, Cambridge CB10 1HH, United Kingdom;; ‡ Department of Haematology, University of Cambridge, Cambridge CB2 0PT, United Kingdom;; § Wellcome Trust–Medical Research Council Cambridge Stem Cell Institute, Cambridge CB2 1QR, United Kingdom;; ¶ Department of Infection, Immunity and Cardiovascular Disease, Medical School, University of Sheffield, Sheffield S10 2RX, United Kingdom;; ‖ Bateson Centre, University of Sheffield, Sheffield S10 2TN, United Kingdom; and; # Scottish Fish Immunology Research Centre, School of Biological Sciences, University of Aberdeen, Aberdeen AB24 3UU, United Kingdom

## Abstract

CD4^+^ T cells are at the nexus of the innate and adaptive arms of the immune system. However, little is known about the evolutionary history of CD4^+^ T cells, and it is unclear whether their differentiation into specialized subsets is conserved in early vertebrates. In this study, we have created transgenic zebrafish with vibrantly labeled CD4^+^ cells allowing us to scrutinize the development and specialization of teleost CD4^+^ leukocytes in vivo. We provide further evidence that CD4^+^ macrophages have an ancient origin and had already emerged in bony fish. We demonstrate the utility of this zebrafish resource for interrogating the complex behavior of immune cells at cellular resolution by the imaging of intimate contacts between teleost CD4^+^ T cells and mononuclear phagocytes. Most importantly, we reveal the conserved subspecialization of teleost CD4^+^ T cells in vivo. We demonstrate that the ancient and specialized tissues of the gills contain a resident population of *il-4/13b*–expressing Th2-like cells, which do not coexpress *il-4/13a*. Additionally, we identify a contrasting population of regulatory T cell–like cells resident in the zebrafish gut mucosa, in marked similarity to that found in the intestine of mammals. Finally, we show that, as in mammals, zebrafish CD4^+^ T cells will infiltrate melanoma tumors and obtain a phenotype consistent with a type 2 immune microenvironment. We anticipate that this unique resource will prove invaluable for future investigation of T cell function in biomedical research, the development of vaccination and health management in aquaculture, and for further research into the evolution of adaptive immunity.

## Introduction

CD4^+^ T cells are pivotal for mounting an effective immune response, but also in maintaining peripheral tolerance and preventing immunopathology, and increasing evidence suggests their central involvement in tumor progression (1–4). Such a crucial function might intuitively imply an ancient and conserved role for these cells in the history of adaptive immunity. However, our knowledge of the evolution of these cells is remarkably limited. Teleost fish represent one of the most ancient and diverse taxa to possess a system of adaptive immunity driven by B and T lymphocytes, and they have therefore been the focus of considerable attention in recent years (5–7). However, despite significant progress, basic questions remain as to the development and function of fish CD4^+^ cells.

In mammals, naive CD4^+^ T cells are activated by Ag stimulation and differentiate into specialized Th effector subsets under the direction of the cytokine microenvironment and the induction of lineage-determining transcription factors (TFs). Two major Th subsets, Th1 and Th2, elicit cell-mediated immunity to intracellular pathogens and humoral immunity to extracellular pathogens, respectively. Differentiation of Th1 cells is promoted by IL-12 inducing the expression of T-bet and STAT4; Th1 cells elaborate IFN-γ that stimulates macrophages and CTLs (3). Differentiation of Th2 cells is promoted by IL-4 induction of GATA3 and STAT6; Th2 cells produce IL-4, -5,-6, -9, -10, and -13 that stimulate B cells, eosinophils, and mast cells (3). Since the discovery of Th1 and Th2 cells, the complexity and plasticity of the Th system has become increasingly clear and additional subpopulations have subsequently been identified. These include Th3, Th9_,_ Th17_,_ Th22, Tr1, and T follicular helper cells with distinct TF and cytokine expression profiles that specialize in mounting an immune response to neutralize particular pathogens (e.g., helminths or fungi) and that are enriched at certain anatomical sites (2). Another important class of CD4^+^ T cells, regulatory T cells (Tregs), typically characterized by expression of the TF Foxp3 and expression of the anti-inflammatory cytokines IL-10 and TGF-β, are essential for resolution of an immune response and for maintaining peripheral tolerance (1). Cytotoxic CD4^+^ T cells have also been described (8). Additionally, the CD4 protein, which acts as a critical coreceptor molecule in T cells, is also known to be expressed in certain populations of mononuclear phagocytes (MNPs) in the mouse, including a specialized population within the thymus (9, 10). Moreover, CD4 expression is widespread among human MNPs (11), and although the function of CD4 in MNPs is not well understood, recent evidence has indicated a critical role in driving differentiation of human monocytes into inflammatory macrophages (12, 13).

There is currently no definitive evidence that Th subsets truly exist in fish, and little is known about the evolutionary origin of these cells. Fish genomes usually contain multiple *cd4*-like paralogs designated *cd4-1* and *cd4-2,* and although the function of the latter gene is currently unknown, we and others have reported evidence that *cd4-1* encodes a canonical CD4 molecule (14–16). Notably, the CD4-1 and CD4-2 proteins of various fish species differ in terms of Ig domain structure, with CD4-1 exhibiting a four Ig domain structure comparable to that of mammalian CD4 (17, 18). In contrast, CD4-2 proteins contain fewer (2, 3) Ig domains, and the functional significance of this is currently unclear. Interestingly, a recent study of the rainbow trout (*Oncorhynchus mykiss*) identified a minor population of T cells that express only CD4-2, and this side population was found to be less proliferative and restricted in TCR repertoire (15). However, evidence suggests that these proteins are widely coexpressed in the T cells of both rainbow trout and zebrafish (14, 15).

Although CD4^+^ cells have not yet been extensively characterized in bony fish, T-bet, GATA, and STAT family TFs (as well as Foxp3) (19) are represented in most teleost genomes, as are many ILs (6). Moreover, CD4^+^ cells isolated from zebrafish, Japanese pufferfish (*Takifugu rubripes*), sea bass (*Dicentrarchus labrax*), and the common carp (*Cyprinus carpio*) treated with nonspecific immunostimulants express signature Th1, Th2, and Th17 cytokines (19–22). Additionally, adoptive transfer experiments using the ginbuna crucian carp (*Carassius auratus langsdorfii*) have provided some evidence that CD4^+^ T cells provide helper function (23, 24). Recent studies employing antisera to zebrafish and rainbow trout CD4-1 proteins have indicated that fish CD4-1^+^ T cells primed and boosted with Ag, or from hosts infected with pathogenic bacteria, will express Th1-, Th2-, and Th17-associated TFs and cytokines (14–16). Thus, in broad terms, it appears likely that T cell function as understood in mammals had already evolved in bony fish. However, the immune responses described in these experiments were heterogeneous and therefore unable to identify CD4^+^ T cells biased toward a particular Th phenotype. Exploring both the conservation and distinctiveness of teleost T cells is of profound interest not just for the evolution of these versatile cells, but also for the application of zebrafish as a model for biomedical research as well as for research into health management of valuable fish species in commercial aquaculture.

The zebrafish is unique among teleost species in readily facilitating genetic manipulation, combined with the ability to perform high-resolution imaging of immune cells in vivo. To promote the further characterization of teleost CD4-1^+^ T cells, we have created a transgenic zebrafish line that faithfully reports expression of CD4-1. The reporter construct facilitates visualization of CD4-1^+^ cell behavior in vivo as well as their ready isolation from tissues by flow cytometry. Using this model we provide further evidence that CD4-1^+^ macrophages are an ancient cell type present in lower vertebrates. We define distinct populations of CD4-1^+^ MNPs resident in the epidermis and gut, as well as a specific population of thymus-resident CD4-1^+^ MNPs that is conserved in mammals. We examine dynamic cell–cell interactions between T cells and perithymic MNPs during thymic egress. Most notably, for the first time to our knowledge, we establish the presence of *il-4/13b*–expressing, Th2-like cells resident in the specialized tissue of the gill. We also demonstrate the existence of a conserved population of *foxp3a*-expressing Treg-like cells resident in the gut mucosa. Finally, we employ an established transgenic cancer model to show that, as in mammalian tumors, zebrafish CD4-1^+^ T cells will infiltrate melanoma tumors. We show that infiltrating CD4-1^+^ cells display a phenotype consistent with a type 2 immune microenvironment. These data suggest that the ability of CD4-1^+^ T cells to orchestrate immunity through subspecialization would likely have been observed in the common ancestor of tetrapods and teleosts. Conservation of these roles illustrates the great potential of the zebrafish system for both biomedical research and further investigation into the origins of adaptive immunity.

## Materials and Methods

### Zebrafish

The *Tg*(*cd4-1:mCherry*) transgenic was generated as described below on the *casper* mutant background to facilitate imaging and observation (25). The *Tg*(*fms:GFP*)*^SH377^* was generated as described below on a *nacre* mutant background. The *lck:GFP* (a gift from Dr. Rui Monteiro), *mhc2dab:GFP* (a gift from Dr. Valerie Wittamer), and *Tg*(*mitfa:eGFP,mitfa:V12RAS*)*^umc1^* transgenic lines have been described previously (26–28), as has the *rag2^E450fs^* mutant line (29).

### Bacterial artificial chromosome recombineering and transgenesis

The *cd4-1* bacterial artificial chromosome (BAC) clone CH73-296E2 (obtained from BACPAC Resources Center, Oakland, CA) and *c-fms* BAC clone HUKGB735K06247Q were modified using the Red/ET BAC recombineering kit (GeneBridges, Heidelberg, Germany) as previously described (30). Briefly, bacteria containing the relevant BAC and recombineering vector (pCS101-BAD-gbaA-tet) were cultured (32°C, 180 rpm) to OD_600_ of 0.6. When the culture reached the desired density, it was divided to two flasks each of 25 ml bacterial culture. To activate the recombineering vector, we added 350 μl of 10% l-arabinose to one of the flasks (induced) or 350 μl of sterile distilled water (uninduced control), which was incubated (37°C, 180 rpm) for 40 min and then cooled on ice for 2 min. Bacteria were then made electrocompetent and transformed with 150 ng of the targeting cassette (iTol2_Kan cassette, *cd4-1:mCherry*, or *c-fms:venus*) using either kanamycin (after Tol2 recombineering, 50 ng/μl) or kanamycin (50 ng/μl) plus spectinomycin (50 ng/μl) (after *cd4-1:mCherry* or *c-fms:Venus*) antibiotic resistance selection and PCR screening of colonies for respective targeting constructs. The mCherry and GFP targeting constructs were assembled using four-fragment multisite gateway technology (Invitrogen, Thermo Fisher Scientific) for recombination into the start codon. The “iTol2_Kan” cassette was a gift from Prof. Stephen Renshaw (University of Sheffield). One-cell-stage embryos were injected with 50 pg of BAC and 50 pg of Tol2 mRNA.

### Flow cytometry and cytology

Single-cell suspensions were prepared from zebrafish organs essentially as described previously (14). Briefly, organs were dissected in to L-15 medium (Life Technologies) with 10% FCS (Sigma-Aldrich). The tissues were pooled from five to six fish and cells were dissociated manually by pushing through a 40-μm pore size cell strainer (BD Falcon) and then further passed through a 50-μm pore size filter (BD Biosciences) to ensure removal of aggregated cells. Cells were centrifuged at 400 × *g* for 5 min at 4°C and resuspended in L-15 media (without phenol red, Life Technologies) with 2% FCS. Dissected intestine and tumor samples were first treated for 1 h at 37°C with Liberase enzyme mixture to facilitate dissociation of cells (Roche, 0.2 U/ml in PBS). Flow cytometry was performed using a FACSAria Fusion flow cytometer (BD Biosciences) and data analyzed using FACSDiva 8.0.1 software (BD Biosciences). For flow cytometry of cells from 20 d postfertilization (dpf) *Tg*(*fms:GFP*)*^SH377^;Tg*(*cd4-1:mCherry*) double transgenics, single-cell suspensions were prepared from individual larvae by manual dissociation in trypsin (Sigma-Aldrich) for 1 h at 37°C. Flow cytometry was performed using an Attune NxT (Applied Biosystems) and data were analyzed using Attune NxT software v2.1. Cytospin and Wright–Giemsa staining was carried out as previously described (31) and cells were imaged using a Zeiss AxioVision microscope.

### Real-time quantitative PCR

RNA was isolated from cells or homogenized tissues using the RNeasy micro kit (Qiagen), including on-column DNase digestion, and stored at −80°C. Reverse transcription was carried out using the ProtoScript II first-strand cDNA synthesis kit (New England Biolabs) with oligo(dT) (deoxythymine) primers. Depending on the number of samples, real-time quantitative PCR (qPCR) was performed either using SYBR Green JumpStart *Taq* ReadyMix (Sigma-Aldrich) and the MX300P system (Stratagene), or using the Biomark HD microfluidic platform (Fluidigm) according to the manufacturers’ instructions, with most data replicated using both methods. Briefly, for Fluidigm Biomark, high-throughput qPCR is performed in two steps. First, target genes are preamplified in a single 14-cycle reaction by combining 25 ng of cDNA with a pooled target primer mix and TaqMan PreAmp Master mix (Applied Biosystems) following conditions recommended by the manufacturer (Fluidigm), and then treated with *ExoI* (New England Biolabs) to remove unincorporated primers. Second, 48 × 48 (samples × primers) qPCR reactions were performed on the Biomark HD dynamic array using EvaGreen for detection and following the manufacturer’s instructions. Ct values were calculated using the system software (Fluidigm real-time PCR analysis version 3). Data were analyzed by the ΔCt method using *bactin* (or *ef1a* where indicated) for normalization [2^−(Ct,^*^gene^* ^− Ct,^*^bactin^*^)^], or the ΔΔCt method using *bactin* and a control sample for normalization. For primer sequences, see Table I.

**Table I.** Primer oligonucleotide sequences

Gene	Primer Sequences
*lck*	F: 5′-CAAACTAGAGCGCAGACTGG-3′
	R: 5′-GGGTGCTGTAGGGACTTCAT-3′
*tcra*	F: 5′-CTTAAAACGTCGGCTGTCCG-3′
	R: 5′-TGAACAAACGCCTGTCTCCT-3′
*mpeg1*	F: 5′-ATGTGGATTCCCCAAACTTCAACT-3′
	R: 5′-TGGTAAATGCCACCAAAGCTAAGA-3′
*c-fms*	F: 5′-GGCCAAAATCTGTGACTTCGGA-3′
	R: 5′-ACCAGACGTCACTCTGAACCG-3′
*cd4-1*	F: 5′-GTGTTTGGACATGCCAGTTG-3′
	R: 5′-AAGCACAGGGAATGCTGACT-3′
*cd8a*	F: 5′-AGGTTGTGGACTTTTCCTCGT-3′
	R: 5′-GGAGCTAGAAGTGGCTGGTG-3′
*ThPok*	F: 5′-CGCCCTTTATTAATGACCCGCG-3′
	R: 5′-AGGCAGCTTGACCTTTCACATG-3′
*cd4-2.1*	F: 5′-GCCTGGACTTGCTGGAGAAATTTT-3′
	R: 5′-AGAACCACAGAAAAGGCTCCTACA-3′
*cd4-2.2*	F: 5′-GGGAAGTTTGTGTGTGAAGTGGAG-3′
	R: 5′-AAACGCACAGTGCGTGTAGATCTG-3′
*runx3*	F: 5′-CAAACTTTCTCTGCTCGGTCCTG-3′
	R: 5′-AATTCTCATCATTTCCCGCCATCA-3′
*eomesb*	F: 5′-AATAACAAGGGCGCGAACATCAA-3′
	R: 5′-AAGATCTGAGTGTTTGAGTCTCGC-3′
*t-bet*	F: 5′-GCAGCTCCAACAACGTAGCA-3′
	R: 5′-ATCCTCCTTCACCTCCACGATG-3′
*gata3*	F: 5′-GGTGAGATGTAGGGAGAGGAAACC-3′
	R: 5′-TGCCCAAGACCTATAACACATCCA-3′
*il-4/13b*	F: 5′-CTGTTGGTACTTACATTGGTCCCC-3′
	R: 5′-AGTGTCCTGTCTCATATATGTCAGGT-3′
*il-4/13a*	F: 5′-GCACTGTATTCGTCTCGGGTTTTA-3′
	R: 5′-TTTTCCCCAGATCTACAAGGAAGA-3′
*ifng1-2*	F: 5′-CCTGGGGAGTATGTTTGCTGTTTT-3′
	R: 5′-GGGTGTGCATTATGTAGCTGAGAA-3′
*foxp3a*	F: 5′-CCGTCACAACACTGCTACATGG-3′
	R: 5′-ACCTTTCCTTCCTTCAACACGC-3′
*il-10*	F: 5′-CTTTAAAGCACTCCACAACCCCAA-3′
	R: 5′-CTTGCATTTCACCATATCCCGCTT-3′
*bactin*	F: 5′-CGAGCTGTCTTCCCATCCA-3′
	R: 5′-TCACCAACGTAGCTGTCTTTCTG-3′

### Tissue preparation, cryosectioning, immunohistochemistry, and in situ hybridization

Dissected gills were fixed in Bouin’s fixative and mounted in 1% low melting temperature agarose (Flowgen). For sectioning, gut was fixed in 4% paraformaldehyde, embedded in 25% fish gelatin/15% sucrose, and sectioned at 20 μm thickness on a Leica 3050 S cryostat. Immunohistochemistry was performed for enhanced GFP or mCherry according to standard protocols using rabbit polyclonal anti-GFP (1:500, Ab290; Abcam), mouse monoclonal anti-mCherry (1:500, Living Colors; Clontech), anti-rabbit Alexa Fluor 488 (1:500; Molecular Probes), and anti-mouse Alexa Fluor 594 (1:500; Molecular Probes). Whole-mount in situ hybridization was carried out as previously described (32). To generate the *cd4-1* probe, an ∼1-kb fragment was cloned by RT-PCR using the following primers: forward, 5′-CGCGTCTCTCTATCAGCAGA-3′, reverse, 5′-CTGTTTGTGTCTGCGGATGT-3′.

### Single-cell whole-transcriptome amplification, data processing, and clustering

Cells were collected from gills and spleen and processed as previously reported (33). Reads were aligned to the zebrafish genome (Ensembl Biomart version 83) combined with the mCherry and 92 External RNA Controls Consortium (ERCC) spike-ins sequences as artificial transcripts and quantified using Sailfish version 0.9.0 (34) with default parameters in paired-end mode (parameter –1 IU). Single cells that expressed <1000 genes or had an ERCC content >60% were excluded from the further analysis. Out of 176 single cells, 99 passed the quality control and were used for further analysis. For each of the 99 cells, counts were converted to counts per million and normalized to account for library size and cell-specific biases using the method proposed by Lun et al. (35) implemented in the *scran* R package (version 1.3.0). Genes that were expressed in fewer than five cells were excluded from further analysis. The technical noise was modeled based on the ERCC counts, and the most highly variable genes were extracted using the *scLVM* R package (version 0.99.2) (36). Principal component analysis was applied to the most variable genes using the implementation of *Seurat* R package (version 1.2.1) (37). Hierarchical clustering using the Euclidean distance and the Ward’s minimum variance criterion was implemented by means of the *pheatmap* R package (version 1.0.8) and applied to the first two principal components (jack straw, *p* < 0.01). To visualize the structure of the data, we performed *t*-distributed stochastic neighbor embedding (*t*-SNE) (38) of the two latent factors into two dimensions. We set the perplexity parameter to 9 and used a fixed random seed to make sure the *t*-SNE plot would be reproducible (parameter k.seed = 1 in the *Seurat* implementation of *t*-SNE). Differential gene expression between each cluster versus the other two was assessed (*p* < 0.01 and *q* < 0.1 [false discovery rate]) using the monocle R package (version 1.99.0) (39). Statistically significant genes for each cluster were further ranked based on the highest to the lowest log_2_ fold change. The top 300 genes were selected from each cluster and the corresponding human orthologs were identified (Ensembl Biomart).

### Live imaging and microscopy

Zebrafish larvae were anesthetized using MS-222 (Sigma-Aldrich) and immobilized in 1% low melting temperature agarose (Flowgen). Time-lapse imaging (one image every 1–2 min) was performed using a Leica SP5 confocal microscope. Z-stacks of ∼40 μm (2–3 μm/Z-slice) were projected onto a single plane. Videos were generated using National Institutes of Health ImageJ. Sections and fixed preparations were imaged using either a Leica SP5 or a Nikon A1R confocal microscope. Fluorescence microscopy was performed using a Leica M205FA stereomicroscope. For imaging of 20 dpf *Tg*(*fms:GFP*)*SH377;Tg*(*cd4-1:mCherry*) compound transgenics, larvae were fixed in 4% paraformaldehyde for 24 h at 4°C, washed in 0.1% Tween 20 PBS, and imaged using a Nikon Ti fluorescence microscope.

### Statistical analysis

Data were analyzed as appropriate using a nonparametric Mann–Whitney *U* test, a one-way ANOVA test, or a Kruskal–Wallis test with a Dunn multiple comparison post hoc test (Prism 6.0; GraphPad Software). In all cases, the significance threshold was set at *p* ≤ 0.05.

## Results

### Generation of a transgenic CD4-1 reporter

**FIGURE 1.**
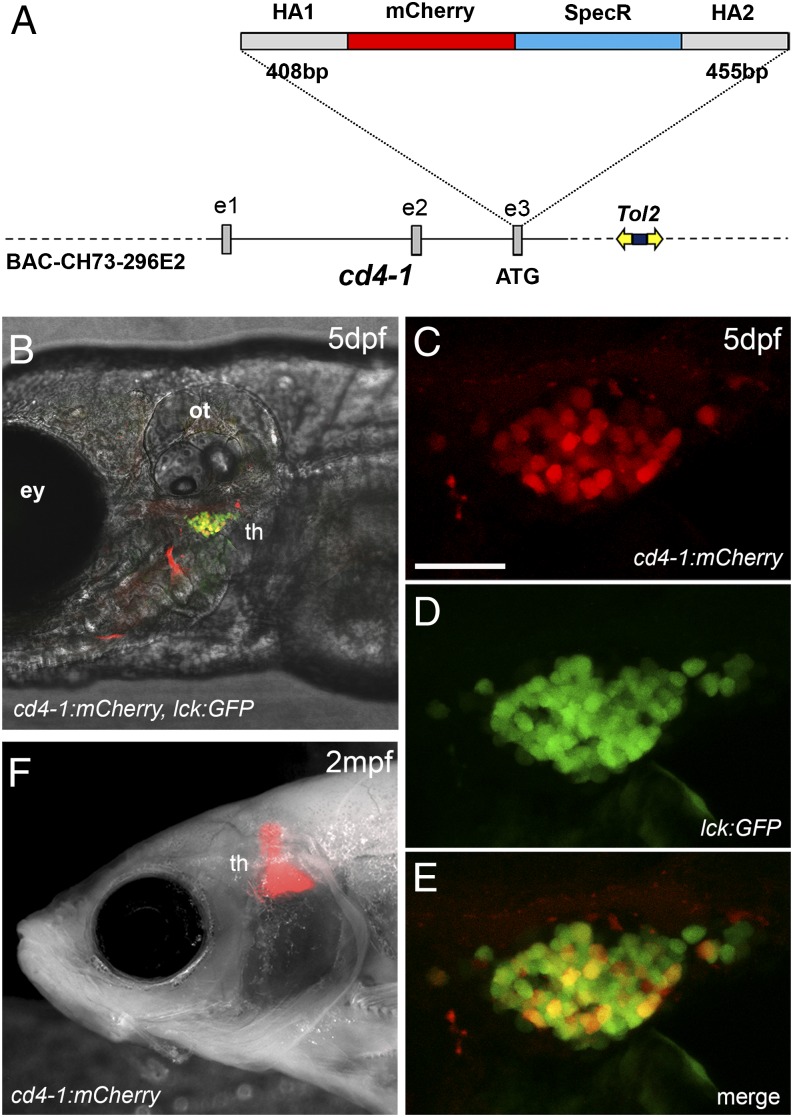
Generation of a *cd4-1* reporter line. (**A**) Schematic illustrating the construction of the transgene. A BAC containing the 5′ region of the *cd4-1* gene was selected and modified by recombination to contain *Tol2* transposable elements with the kanamycin resistance gene (dark blue box) to allow selection. A cassette containing the *mCherry* coding sequence and the spectinomycin resistance gene (*SpecR*) flanked by *cd4-1* homology arms (HA1 and HA2) was then recombined into the *cd4-1* start codon (located in exon 3). (**B**) The *Tg*(*cd4-1:mCherry*) reporter is expressed in the thymus by 5 dpf and colocalizes with the pan–T cell reporter *lck:GFP.* (**C**–**E**) High magnification view of the thymus at 5 dpf revealing CD4-1^+^ (mCherry^+^ GFP^+^) and CD4-1^−^ (GFP^+^ only) T cells. (**F**) Reporter expression in the thymus of adult fish at 2 mo postfertilization in the *casper* mutant background. Anterior is to the left. Scale bar, 10 μm. Denoted features are eye (ey), otic vesicle (ot), thymus (th).

We have previously determined that zebrafish, similar to other teleosts, express two full-length *cd4* paralogs, termed *cd4-1* and *cd4-2.1* (*cd4-2.2*, a third paralog unique to zebrafish, is predicted to lack a cytoplasmic tail or LCK CXC docking motif, and may perform a novel regulatory function) (14). CD4-1 is likely to represent a functional ortholog of mammalian CD4, as it has a closer genomic organization, Ig domain structure, and conserved regulatory regions. To facilitate visualization and isolation of zebrafish CD4-1^+^ cells, we generated a fluorescent transgenic reporter line. To use important transcriptional regulatory elements from the *cd4-1* locus, we used BAC recombineering to integrate mCherry fluorescent protein into the initiation codon of *cd4-1* located in exon 3 (Fig. 1A). We then introduced the recombined BAC into the zebrafish genome by coinjecting it into zygotes together with Tol2 transposase mRNA. Expression of *cd4-1* mRNA was just detectable at 4 dpf, but increased over days 5−6 of development (Supplemental Fig. 1A). In situ hybridization confirmed expression of *cd4-1* mRNA in the thymus by 6 dpf (Supplemental Fig. 1B). Consistent with this, breeding from a *Tg*(*cd4-1:mCherry*) founder resulted in progeny with robust red fluorescence apparent in the thymus by 5 dpf (Fig. 1B–E). Thereafter, mCherry fluorescence persisted in the thymus into adulthood (Fig. 1F), and a robust signal was still apparent in fish over 4 mo old. Breeding allowed us to combine the *Tg*(*cd4-1:mCherry*) construct with the established pan–T cell reporter line, *lck:GFP* (26). Double-positive (mCherry^+^/GFP^+^) cells could be identified in the thymus of *Tg*(*cd4-1:mCherry*)*;lck:GFP* transgenic animals (Fig. 1C–E), consistent with the labeling of a CD4-1^+^ T cell population.

### Morphology, expression characteristics and distribution of *Tg*(*cd4-1:mCherry*)*^+^* cells

**FIGURE 2.**
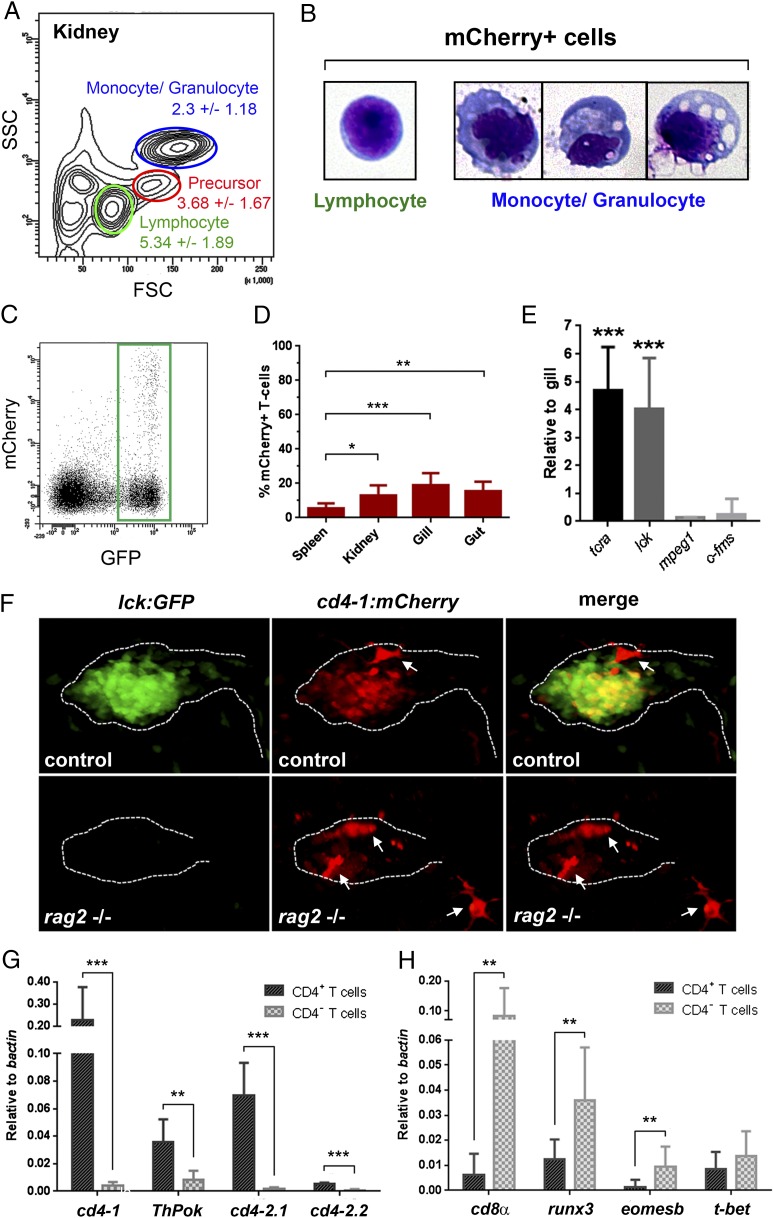
Distribution and gene expression in CD4-1^+^ T cells. Kidney and spleen from adult (aged 3–9 mo) *Tg*(*cd4-1:mCherry*)*;lck:GFP* fish were analyzed by flow cytometry. (**A**) Forward and side scatter profiles (FSC/SSC) indicating the percentage of mCherry^+^ cells present in each gate. (**B**) mCherry^+^ cells isolated from the lymphocyte (left panel) or monocyte/granulocyte (right panel) gate when subjected to Wright–Giemsa stain. (**C**) Lymphocytes were gated for *lck:*GFP^+^ cells. Original magnification ×20. (**D**) Proportion of mCherry^+^ cells among GFP^+^ T cells in adult organs (*n* = 8–11, *p* < 0.05). (**E**) qPCR analysis shows that CD4-1^+^ cells isolated from the lymphocyte gate express *tcr α*-*chain* (*tcra*) and *lck*, but not *mpeg* or *c-fms*. (**F**) *rag2*^−/−^ larvae exhibit a loss of *lck:*GFP^+^ and *Tg*(*cd4-1:*mCherry)^+^ T cells within the thymus compared with control siblings. Thymus-associated *Tg*(*cd4-1:*mCherry)^+^ MNPs are indicated by arrows (images at 7 dpf). Original magnification ×20. (**G** and **H**) qPCR analysis shows that mCherry^+^ T cells express *cd4-1*, *ThPok*, *cd4-2.1*, and *cd4-2.2* (G) and mCherry^−^ T-cells express *cd8a*, *runx3*, *eomesb*, and *t-bet* (H) (*n* = 7, *p* < 0.05). Error bars represent SD. *p < 0.05, **p < 0.01, ***p < 0.001.

To continue our characterization of zebrafish CD4-1^+^ cells, we flow sorted from *Tg*(*cd4-1:mCherry*)*;lck:GFP* double-transgenic zebrafish kidney and spleen using complexity and size to distinguish leukocyte populations (40). As expected, we found that mCherry^+^ cells comprised a small subset (5.34 ± 1.89%) of the lymphocyte gate (Fig. 2A). We also observed an mCherry^+^ population of cells within the monocyte/granulocyte gate (2.3 ± 1.18%), suggesting the existence of CD4-1^+^ MNPs (Fig. 2A). Cytospin and Wright–Giemsa staining confirmed that mCherry^+^ cells isolated from the lymphocyte gate had characteristic lymphocyte morphology (Fig. 2B), whereas mCherry^+^ cells isolated from the monocyte/granulocyte gate demonstrated heterogeneous morphologies typical of MNPs (Fig. 2B). By gating the lymphocytes, we confirmed the existence of (GFP^+^/mCherry^+^) CD4-1^+^ T cells in addition to GFP^+^-only T cells (Fig. 2C). Flow cytometry revealed that CD4-1^+^ T cells comprised between 10 and 20% of T cells isolated from the kidney, gills, and gut of adult zebrafish, but was significantly lower (5.55% ± 2.79) in the spleen (Fig. 2D). qPCR analysis revealed that the CD4-1^+^ T cell population expressed the T cell markers *tcra* and *lck*, but not the macrophage markers *mpeg1* or *c-fms* (Fig. 2E), consistent with their classification as T cells. To further confirm this, we also examined *Tg*(*cd4-1:mCherry*) expression in the *rag2*^−/−^ mutant background, which is known to cause a marked reduction of thymic and mature T cells (29). By 7 dpf the *rag2* mutant homozygous animals demonstrated loss of both *lck:GFP*^+^ and *Tg*(*cd4-1:mCherry*)^+^ T cell populations within the thymus (Fig. 2F). Interestingly, we also noticed that a population of thymus-associated CD4-1^+^ MNPs appeared to be retained in the *rag2* mutant larvae (Fig. 2F, white arrows).

We next examined gene expression in CD4-1^+^ and CD4-1^−^ T cell populations. We confirmed by qPCR that the *cd4-1* gene was expressed strongly in mCherry^+^ T cells, corroborating their identity as CD4-1^+^ T cells (Fig. 2G, Supplemental Fig. 1C). Furthermore, qPCR revealed increased expression of the Th lineage committing TF *ThPok* (*zbtb7b*) (41) in mCherry^+^ T cells and greatly enriched expression of *cd4-2.1* and *cd4-2.2* (Fig. 2G), indicating that the expression of *cd4* paralogs overlaps extensively in zebrafish. Conversely, *cd8α* expression was detected largely in the mCherry^−^ population, suggesting it is composed primarily of CD8^+^ T cells (Fig. 2H). Expression of the *runx3* and *eomesb* TFs was enriched in CD4-1^−^ T cells relative to CD4-1^+^ T cells, consistent with their established roles in the development of mammalian CD8^+^ T cells (42). Both CD4-1^+^ and CD4-1^−^ T cells expressed the *t-bet*/*tbx21* TF as is the case in mammals. Additionally, to provide further detail on the specificity of the reporter, we isolated single mCherry^+^ cells from the spleen and gills of *Tg*(*cd4-1:mCherry*) transgenic animals and subjected them to RNA sequencing (Supplemental Fig. 1D–G). Hierarchical clustering independently confirmed that the cells labeled by the reporter identify as either T cells or MNPs, and it indicated that *cd4-1* expression is considerably higher in the T cell population (Supplemental Fig. 1D–G). Taken together, these data confirm the *Tg*(*cd4-1:mCherry*) transgenic as an effective and accurate reporter of CD4-1^+^ T cells, and they provide further evidence that the transcriptional machinery underlying CD4-1^+^ and CD8^+^ T cell maturation is conserved between mammals and teleost fish.

### Egress of zebrafish CD4-1^+^ T cells and scrutiny by CD4-1^+^ perithymic MNPs

**FIGURE 3.**
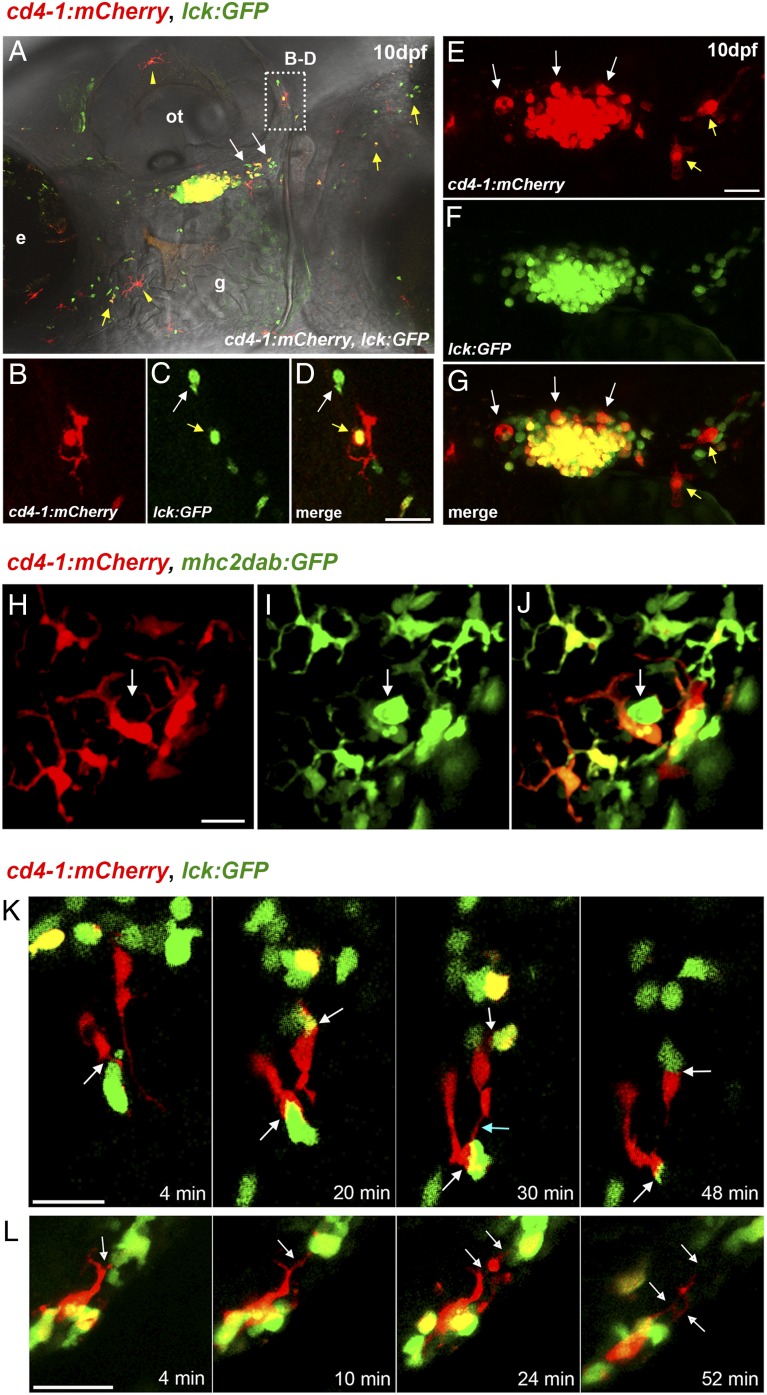
Developmental expression of *Tg*(*cd4-1:mCherry*). (**A**) Composite image of *Tg*(*cd4-1:mCherry*) and *lck:GFP* expression at 10 dpf. T cells can be seen migrating out of the thymus (white arrows). CD4-1^+^ and CD4-1^−^ T cells are visible in the integument (yellow arrows), as is a population of skin-resident MNPs (yellow arrowheads). (**B**–**D**) High magnification image from the otic region in (A) showing a CD4-1^−^ T cell (white arrow) and a CD4-1^+^ T cell (yellow arrow) in close contact with an MNP (large red cell). (**E**–**G**) Intrathymic MNPs (white arrows) and perithymic MNPs (yellow arrows) can be identified by 10 dpf. (**H**–**J**) Images of *Tg*(*cd4-1:mCherry*)*;mhc2dab:GFP* MNPs located in the skin of 10 dpf zebrafish. (H–J) The *Tg*(*cd4:mCherry*)^+^ MNPs exhibit a noticeably more dendriform morphology than do the *mhc2dab:GFP*^+^ single-positive cells (I and J, white arrows). (**K** and **L**) Live time-lapse imaging of the perithymic region at 10–14 dpf. (K) Extended synapsing between CD4-1^+^ MNPs and T cells (white arrows). MNPs are also seen to connect temporarily (blue arrow). (L) Cytoplasmic tethering (white arrows) occurs between an MNP and T cell. Scale bars, 20 μm. Denoted features are eye (e), otic vesicle (ot), gill (g).

In addition to facilitating studies of CD4-1^+^ T cells, our initial observations indicated the intriguing possibility of an ancient evolutionary origin for CD4-1^+^ MNPs (15). We first examined the developmental expression of *Tg*(*cd4-1:mCherry*);*lck:GFP* double reporter and noted that by 10 dpf, thymic egress by both CD4-1^+^ and CD4-1^−^ T cells could be readily observed, with a steady stream of cells emanating from the caudal region of the thymus and entering the circulation, most notably migrating adjacent to the posterior region of the otic vesicle (Fig. 3A–D). This region becomes densely populated by 20 dpf (Supplemental Fig. 2A, 2E–G). Interestingly, we observed clear evidence of T cells resident in the periphery by 10 dpf, most notably in the integument (Fig. 3A, yellow arrows; Supplemental Fig. 2B–D). Additionally, thymus-resident MNPs (mCherry^+^/GFP^−^) are well established by 10 dpf intercalating between the densely arranged thymocytes (Fig. 3E–G). At this stage we also observed the emergence of a population of perithymic macrophages that appeared to be located along the corridor of egress, apparently making regular contact with migrating T cells (Fig. 3E–G, yellow arrows). In parallel, we also noted the emergence of a skin-resident population of MNPs (Fig. 3A, yellow arrowheads), which expands greatly in later development and had formed an extensive network of cells by adult stages (Supplemental Fig. 2H, 2I). To characterize the MNP population further, we combined *Tg*(*cd4-1:mCherry*) with the previously described *mhc2dab:GFP* reporter line, which labels MNPs, including those of the skin (28). We observed two distinct populations of MNPs in the skin, a *Tg*(*cd4-1:mCherry*) (mCherry^+^GFP^+^)–expressing population, and a second group of MNPs that express only the *mhc2dab:GFP* reporter (mCherry^−^GFP^+^), which appear markedly less dendriform (Fig. 3H–J). Additionally, we generated a *c-fms:GFP* transgenic line; crossing this line with *Tg*(*cd4-1:mCherry*) revealed three distinct MNP populations (mCherry^+^GFP^−^, mCherry^+^GFP^+^, and mCherry^−^GFP^+^) in the skin and gut (Supplemental Fig. 3). Flow cytometry demonstrated that ∼20% of *c-fms:GFP*^+^ cells were also *Tg*(*cd4-1:mCherry*)^+^ (Supplemental Fig. 3C–E; median, 22.7%). Conversely, ∼10% of total *Tg*(*cd4-1:mCherry*)^+^ cells were also *c-fms:GFP*^+^ (Supplemental Fig. 3F–H; median, 12.5%).

The ability to visualize and distinguish CD4-1^+^ T cell, CD4-1^−^ T cell, and MNP populations in the *Tg*(*cd4-1:mCherry*)*;lck:GFP* reporter provides an opportunity to examine interactions between immune cells in vivo. We performed live time-lapse microscopy at the 10–14 dpf stage, focusing on the disseminating stream of T cells and resident MNPs in the perithymic region (Fig. 3K, 3L). Live imaging revealed the dynamic nature of interactions between living leukocytes, with resident “sentry”-like MNPs making frequent, intimate, and dynamic physical contacts with migrating T cells. MNPs often form extended membrane synapses with both CD4-1^+^ and CD4-1^–^ T cells while in some cases forming tethers with neighboring MNPs (Fig. 3K, Supplemental Video 1). Additionally, we observed that T cells were often detained by the long tether-like dendritic processes emanating from resident MNPs (Fig. 3L, Supplemental Video 2). Interactions occurred during many minutes and we noted that MNPs frequently contained discrete beads of GFP^+^ cytoplasm.

### Differentiation of tissue-resident CD4-1^+^ T cells indicates extensive subspecialization—a conserved Th2-like phenotype in the gill mucosa

It is currently unclear whether CD4^+^ T cell subsets equivalent to Th1, Th2, and Tregs are present in fish, and the functional relationships of key signature cytokines have yet to be determined. Previous attempts to address this issue by challenging immune cells with pathogens or immunostimulants have generated largely heterogeneous responses (14, 15, 19–24). However, we were interested by the observation that in the steady-state certain tissues or organs maintain immune-biased microenvironments, with significant implications for the administration of therapeutic agents. For example, the gill tissue has been shown in various fish species to constitutively express *Gata3* and *IL-4*–related cytokines, suggesting a Th2-biased microenvironment, although in all cases the source of this expression was unknown (43–47). The *Tg*(*cd4-1:mCherry*) reporter line provides a unique opportunity to explore the conservation and distinctiveness of fish Th cell differentiation within such an environment. Thus, to search for fish Th2 cells, we examined CD4-1^+^ T cell populations of the kidney and spleen, gills, and gut.

**FIGURE 4.**
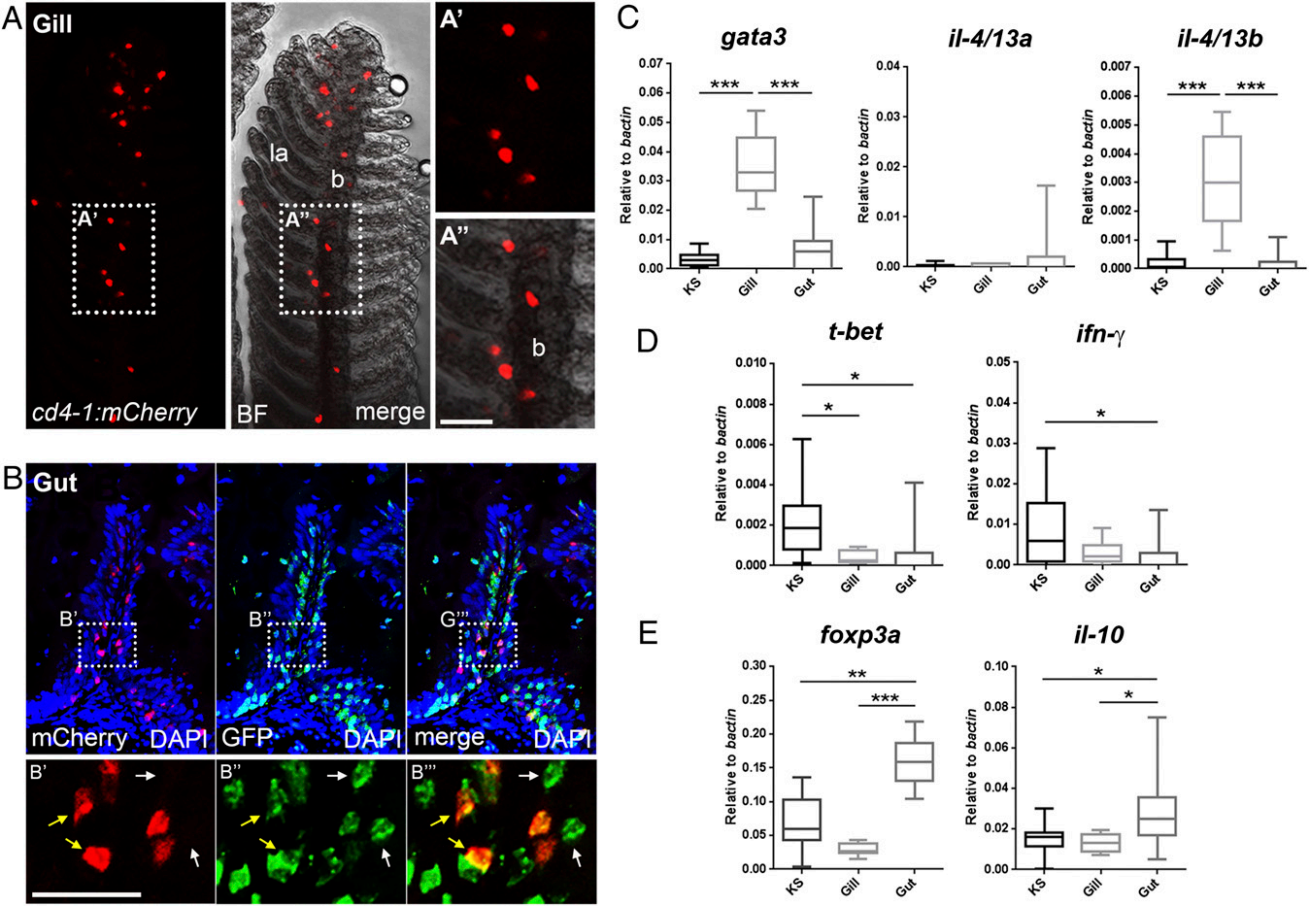
Differentiation of tissue-resident CD4-1^+^ T cells. (**A**) Gill preparation confirming resident population of CD4-1^+^ cells (BF, bright field; la, lamella; b, branchial filament). (**B**) Cryosection and immunostain showing an intestinal villus populated by both CD4-1^+^ (yellow arrows) and CD4-1^−^ (white arrows) T cells. (**C**–**E**) qPCR analysis of CD4-1^+^ T cells isolated from kidney and spleen (KS), gill, and gut. (C) Expression of *gata3* and *il-4/13b*, but not *il-4/13a*, is significantly enhanced in CD4-1^+^ T cells of the gills. (D) Expression of *t-bet* and *ifn-γ* is significantly lower or unaltered in CD4-1^+^ T cells of the gill or gut compared with KS. (E) CD4-1^+^ T cells of the gut show significantly enhanced expression of *foxp3a* and *il-10*. Error bars represent SD; *n* = 9–12, *p* < 0.05. Scale bars, 20 μm. *p < 0.05, **p < 0.01, ***p < 0.001.

We initially sought to confirm the presence of tissue-resident CD4-1^+^ T cell populations in the gill and gut of adult zebrafish. Consistent with this, we detected CD4-1^+^ T cells studding the epithelium of the branchial filaments and occasionally in the lamellae epithelium (Fig. 4A). Cryosectioning of the intestine revealed that CD4-1^+^ and CD4-1^−^ T cells are abundant in the lamina propria of the gut mucosa and are dispersed among overlying epithelial cells (Fig. 4B). We next isolated CD4-1^+^ T cells from the kidney and spleen, gills, and gut and performed qPCR analysis. Compared to cells sourced from other tissues, CD4-1^+^ T cells isolated from gills expressed significantly higher levels of the Th2 signature genes *gata3* and *il-4/13b* (Fig. 4C). In contrast, expression of the Th1 signature genes *t-bet* and *ifn-γ* remain low or significantly reduced (Fig. 4D). Interestingly, we found that the expression of *il-4/13a* remains low in gill-resident CD4-1^+^ T cells (Fig. 4C), supporting the notion that the IL-4/13 paralogs have distinctive roles in teleost fish (47). Previous studies in other fish species had noted constitutive expression of the *il-4/13a* paralog (45, 47). Consistent with this, in total gill tissue, we noted that *il-4/13a* and *il-4/13b* were expressed at similar levels (Supplemental Fig. 4A). We therefore compared gill-resident CD4-1^+^ T cells to total gill tissue from the same animal (Supplemental Fig. 4B). CD4-1^+^ T cells of the gill are greatly enriched for the expression of *il-4/13b* and significantly depressed for the expression of *il-4/13a*, confirming that CD4-1^+^ T cells are not the source of this cytokine. Additionally, we found that CD4-1^+^ cells isolated from the gut displayed a contrasting gene expression signature, with significant upregulation of the Treg signature genes *foxp3a* and *il-10* (Fig. 4E). The presence of a CD4-1^+^ *foxp3a*, *il-10*–expressing population in the gut would appear to mirror the extensive and essential population of Tregs found in the mammalian intestine (48).

**FIGURE 5.**
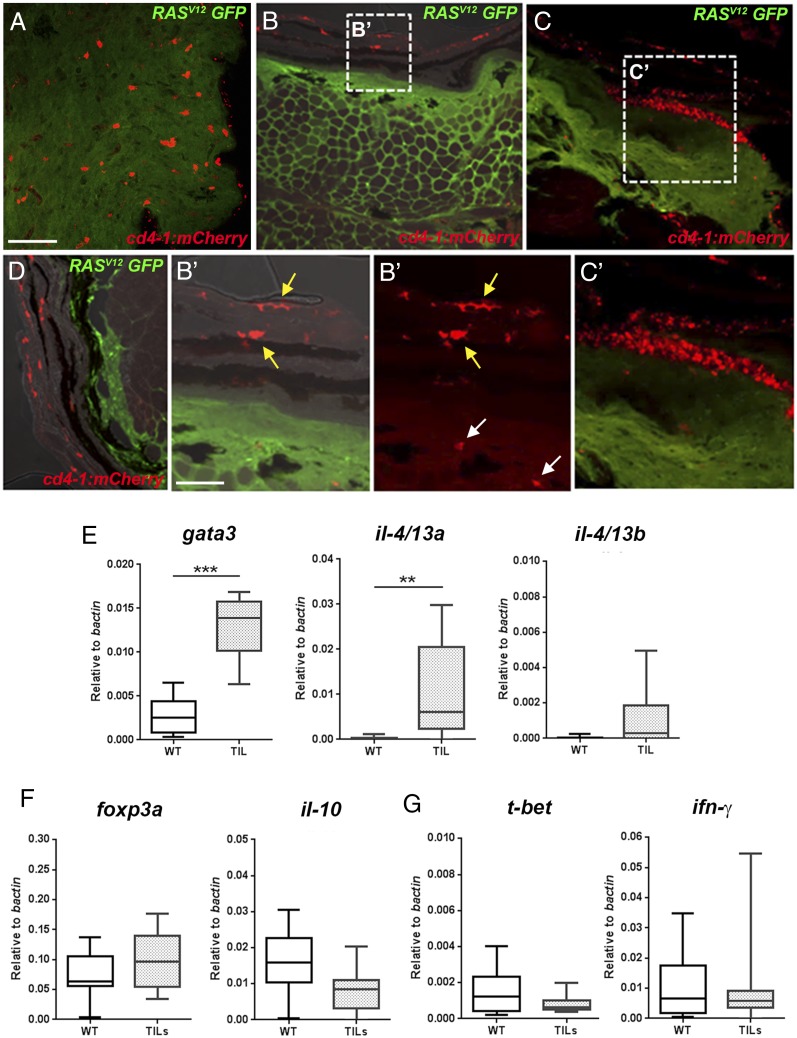
Differentiation of tumor infiltrating CD4-1^+^ T cells. (**A**–**D**) Cryosection and immunostain of zebrafish tumors from animals carrying the *Tg*(*cd4-1:mCherry*) and *mitfa:RAS*^V12^ (GFP labeled) transgenes. (A) Densely infiltrated tumor showing CD4-1^+^ cells (red) and tumor cells (green) (Scale bar, 50 μm). (B and B′) Tumor with few infiltrating CD4-1^+^ cells shown (white arrows in B′). CD4-1^+^ MNPs can be seen in the overlying epidermis (yellow arrows in B’). (C and C′) Clustering of immune cells in adjacent skin. (D) A melanoma lesion with no detectable CD4-1^+^ infiltrate. (**E**–**G**) qPCR analysis showing gene expression in CD4-1^+^ TIL T cells compared with wild-type (WT) T cells from the kidney and spleen. (E) CD4-1^+^ TILs significantly upregulate the expression of *gata3* and *il-4/13a*, but not *il-4/13b*. (F and G) The expression of *foxp3a*, *il-10* (F), and *t-bet* or *ifn-g* (G) is not significantly altered in CD4-1^+^ TILs. Error bars represent SD; *n* = 9–11, *p* < 0.05. **p < 0.01, ***p < 0.001.

We next examined CD4-1^+^ T cells in zebrafish where the immune system had been challenged by the development of melanoma tumors (27) (Supplemental Fig. 4C). Tumors represent a highly immunomodulatory tissue microenvironment, which, although variable, are generally thought to maintain a type 2–biased milieu favoring Th2 or Treg phenotypes and the suppression of Th1 differentiation and the recruitment of CD8^+^ T cells (4, 49). As expected, initial analysis of zebrafish tumors indicated significantly reduced expression of the pan–T cell marker *lck* as well as *cd8α* and *cd4-1* (Supplemental Fig. 4D), implying an overall reduction of T cells during the progression from radial growth phase melanoma to nodular tumors (50). We noticed that a subset of zebrafish tumors expressed high levels of *il-4/13a* and, to a much lesser extent, *il-4/13b* (Supplemental Fig. 4E), consistent with the recent observation that these cytokines have anti-inflammatory activity (47). To confirm that CD4-1^+^ T cells infiltrate melanoma in zebrafish, we initially performed cryosectioning of tumors. We identified lesions containing extensive populations of infiltrating CD4-1^+^ cells (Fig. 5A) (in humans referred to as “brisk” tumors), in addition to lesions with little (Fig. 5B, 5B′) or no (Fig. 5D) infiltrate. We next isolated CD4-1^+^ T cells from the tumors (tumor-infiltrating lymphocytes [TILs]) and compared gene expression to wild-type CD4-1^+^ T cells isolated from the kidney and spleen. Intriguingly, CD4-1^+^ tumor-infiltrating lymphocytes also expressed significantly enhanced levels of *gata3* along with the alternative Th2-associated cytokine *il-4/13a*, but not *il-4/13b*, in marked contrast to the phenotype observed in the gills (Fig. 5D). Consistent with this, there was no significant enrichment for the expression of the Th1 markers *t-bet* or *ifn-γ*, or indeed the Treg genes *foxp3a* or *il-10* (Fig. 5F, 5G).

Taken together, these data demonstrate three distinct polarized populations of CD4-1^+^ T cells with distinctive gene expression signatures. The gills, gut, and melanoma tumors each harbor populations of differentiated CD4-1^+^ T cells with the hallmarks of Th2 or Treg-like cells. To our knowledge, these data are the first in vivo evidence for Th2 or Treg-like cells in a nonmammalian species.

## Discussion

Adaptive immunity has been proposed as one of the key evolutionary innovations in the emergence of vertebrates (51). In this study, we have used zebrafish as a model teleost to expand our understanding of the biology and evolution of CD4-1^+^ T cells and CD4-1^+^ MNPs. We have begun to image and characterize the development of CD4-1^+^ leukocytes and present in vivo evidence for the differentiation and subspecialization of CD4-1^+^ T cells. We also described specific populations of CD4-1^+^ MNPs suggesting that this cell type had already emerged in early vertebrates.

We chose to exploit the *Tg*(*cd4-1:mCherry*) transgenic to look for evidence of subspecialization of CD4-1^+^ T cells in zebrafish. It has so far remained unclear whether Th subsets equivalent to the Th1, Th2, and Treg cells of mammals are present in fish; however, a body of evidence has emerged in recent years that appears, at least broadly, to support the conservation of polarized type 1 and type 2 immune responses. Considerable effort has been made to identify homologs of relevant cytokines and TFs (6), while key effector cells such as CD8^+^ cytotoxic T cells and NK cells (Th1), mast cells, and eosinophils (Th2) have been identified in teleosts (52–55). Moreover, evidence has begun to emerge for polarization of M1- and M2-like macrophages (56, 57). However, the involvement of CD4-1^+^ T cells and the extent of their conservation have not yet been clarified. Recent studies employing antisera to CD4-1 in zebrafish and rainbow trout have shown that stimulation with Ag or infection results in the expression of relevant cytokines and TFs, but they were unable to identify cell populations skewed toward a particular Th phenotype (14–16). In a search for differentiated zebrafish Th cells, we speculated that the immune microenvironment of certain organs and tissues might be skewed toward a particular phenotype in the steady-state. Organs such as the gills or intestine are constantly exposed to foreign Ags, including an extensive local microbiota, and must strike a tightly controlled balance between immunity and tolerance. It is well known that the mammalian gut contains an extensive population of Tregs, which are required to prevent autoimmunity (48). Previous studies had observed the constitutive expression of the putative Th2 markers *Gata3* and *IL-4/13A* from the gill of teleost species, suggesting the maintenance of a type 2–like immune milieu. To look for Th2-like cells we therefore examined gene expression in CD4-1^+^ T cells from the gills and compared them to those isolated from the gut and kidney and spleen. We found that gill-resident CD4-1^+^ T cells were strongly enhanced for *gata3* and *il-4/13b* expression, indicating the presence of a novel population of teleost Th2-like cells. In contrast, the CD4-1^+^ T cells of the gut were enriched for *foxp3a* and *il-10*, suggesting that they are skewed toward a Treg-like phenotype. In both cases the Th1-associated genes *t-bet* and *ifn-γ* were expressed only at low levels.

The observation that the Th2-like population of the gills expresses *il-4/13b* and not *il-4/13a* may be highly significant in beginning to resolve the functional relationship of the teleost IL-4/13–related cytokines. It had previously been speculated that IL-4/13A might represent the functional ortholog of mammalian IL-4, based primarily on the observation of higher constitutive gene expression in certain lymphoid organs and the presence of a Gata3 binding site in the prospective regulatory sequence (58, 59). However, a recent in-depth study of IL-4/13 paralogs in trout indicated that whereas *IL-4/13A* is robustly expressed in various organs, including the gill, the *IL-4/13B1* and *IL-4/13B2* (paralogs apparently derived from an additional genome duplication event specific to salmonids) genes are more responsive to viral or parasitic infection in vivo (47). Such observations are entirely consistent with our in vivo data that indicate that CD4-1^+^ T cells are the source of *il-4/13b*, but not *il-4/13a*, in the gills. Moreover, in carp a Th2-like cell line clone has been reported to express *IL-4/13B*, but not *Il-4/13A*, following PHA treatment (22). It will be interesting in future studies to explore the cellular source of *il-4/13a* expression in the gills and whether this cytokine is required for the differentiation or recruitment of the *il-4/13b*–expressing T cells. In any case, the identification of a Th2-like population of cells within the ancient and specialized tissue of the gills poses interesting questions as to the evolutionary origin of vertebrate type 2 immunity. Further comparative studies of the gills in early vertebrate taxa might shed light on the origin of this type 2 immune niche.

To further our analysis, we examined the CD4-1^+^ T cells infiltrating zebrafish melanoma tumors. For obvious reasons, human tumors represent some of the most keenly studied immune microenvironments. Although heterogeneous, developing tumors must evade eradication by hostile inflammatory effector cells and regularly enforce a type 2–biased milieu featuring infiltration by Th2 cells or immunosuppressive Tregs (4, 49). In recent years, zebrafish models of human cancer, including melanoma, have proven highly informative with extensive phenotypic conservation (27, 60). However, this is the first characterization, to our knowledge, of the immunological phenotype of a zebrafish tumor model. We showed that zebrafish tumors are infiltrated by CD4-1^+^ cells, and that these are enhanced for the expression of *gata3* and *il-4/13a*, but not for *il-4/13b*. These results support the view that these cytokines have discrete functions in teleost immunity (47). However, the identification of a population of *il-4/13a*–expressing T cells, in contrast to cells resident in the gill, raises the intriguing possibility of an alternative Th2-like phenotype. Certainly, heterogeneity of Th2 cells has been observed in mammals, with reports of inflammatory and noninflammatory Th2 cells (61). The infiltration of developing zebrafish tumors by CD4-1^+^ T cells implies a significant degree of functional conservation. However, the future challenge will be to explore the extent of functional homology between the cell populations described in this study and the equivalent cells of mammals, such as whether they respond to similar challenges and provide genuine helper or regulatory functions. The ongoing development of zebrafish models of infectious disease (62–66) and inflammatory disorders (67, 68) coupled with novel genetic tools (such as immune-compromised fish) (69) presents an exciting opportunity to dissect these questions in detail.

In addition to T cells, the *Tg*(*cd4-1:mCherry*) reporter also identifies significant subpopulations of MNPs, including those associated with the thymus, and an extensive network of macrophage-like cells in the skin and gut. Taking advantage of the unique features of zebrafish, we showed that combination of the *Tg*(*cd4-1:mCherry*) reporter with the MNP reporters *c-fms:GFP* and *mhcIIdab:GFP* allows further visual resolution of distinct MNP subpopulations, providing a potential facility for the future characterization of MNPs in the adult fish. CD4^+^ macrophages are prevalent in human biology, yet studies of the role of CD4 in MNPs are surprisingly limited. It has been proposed that CD4 acts as a coreceptor in macrophages, and some interesting work has suggested CD4 to be a key component of the mechanism of HIV infection (12, 13). In the mouse and rat, CD4 is expressed only by specific populations of MNPs, including those within the thymus (9, 10). Our data suggest that this population is conserved in zebrafish, and we have identified a novel population of perithymic MNPs that scrutinize T cells during thymic egress. Using live cell imaging, we were able to document dynamic interactions and extended cell contacts between CD4-1^+^ T cells and MNPs in the perithymic region. The identification of CD4-1^+^ MNPs in teleosts implies that these cells have an ancient evolutionary origin. The observations presented in the present study are satisfyingly complemented by the recent discovery of CD4-1^+^ macrophages in the spleen and kidney of rainbow trout (15). Interestingly, this study also suggested that CD4-1^+^ macrophages may be among the most phagocytic of teleost MNPs. Comparative studies have the potential to highlight key features that have been missed in traditional models (5). We suggest that the discovery that CD4^+^ MNPs are present in lower vertebrates might justify further attention to the role of CD4 in innate immune cells.

Our understanding of adaptive immunity in teleosts lags far behind our state of knowledge for mammals. Nonetheless, this gap is likely to narrow rapidly as technologies allowing in-depth molecular analysis of individual cells are brought to bear. We propose that the zebrafish model has the potential to drive progress in the field of fish immunology. In time, we envisage using zebrafish to learn more about lymphocyte homing, cell–cell interactions, and developmental plasticity where the rapid generation time, facility for genetic manipulation, and visualization of single cells in situ will be enormously beneficial.

## Supplementary Material

Data Supplement
