# Supplementary material for: CD4-Transgenic Zebrafish Reveal Tissue-Resident Th2- and Regulatory T Cell–like Populations and Diverse Mononuclear Phagocytes
Source: J Immunol. 2016 Sep 30;197(9):3520–30. doi: 10.4049/jimmunol.1600959 (PMC5073357; doi:10.4049/jimmunol.1600959)
Supplement: Data Supplement [file JI_1600959.zip › JI_1600959_Supplemental_Figures_1.pdf]

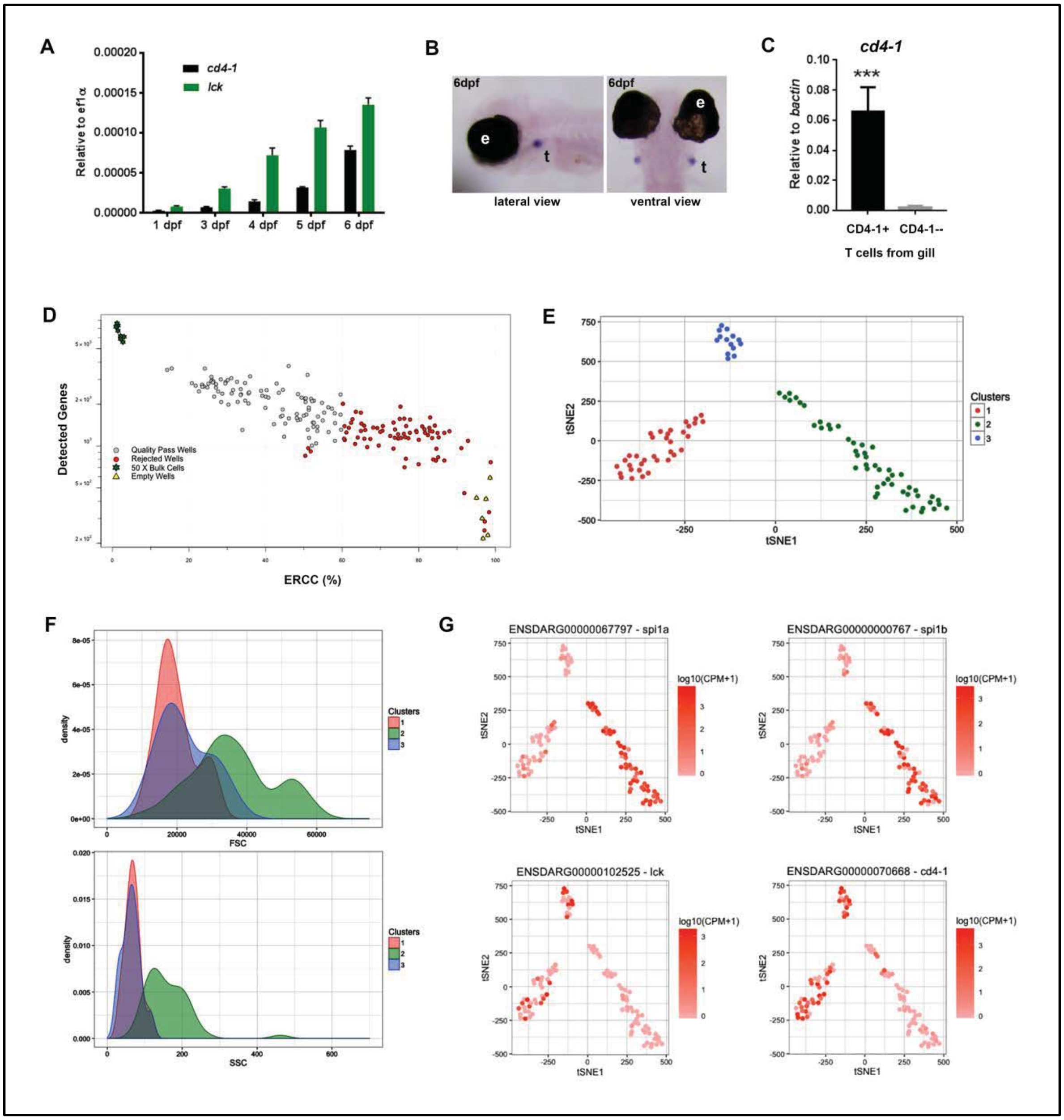

Supplemental Fig. 1. Expression of *cd4-1* mRNA in embryo and adult populations. (**A**) The *cd4-1* transcript is weak but detectable by Q-PCR at 4dpf and increases steadily at 5dpf and 6dpf. The expression pattern correlates with that of the *lck* gene, which is expressed in all T cells. (**B**) *In situ* hybridisation to 6dpf larvae showing expression of *cd4-1* within the thymus (t = thymus, e = eye) (**C**) Q-PCR analysis of T cells extracted from the gills of *Tg[cd4-1mCherry]*, *lck:GFP* double transgenic fish. Expression of *cd4-1* is high in mCherry$^+$ (CD4-1$^+$) T cells and low in mCherry$^-$ (CD4-1$^-$) T cells. (**D-G**) Single cell gene expression analysis of *Tg[cd4-1:mCherry]* expressing leukocytes. (**D**) Two 96-well plates were preloaded with lysis buffer. In each plate we sorted 88 single cells, leaving three wells empty (i.e without cells) and five wells with 50 cells per plate. Following sequencing and quality control, cells (data points) with high ERCC content or with few reads and few genes were removed from further analysis (red dots). As expected wells without cells (yellow triangle) have ERCC content equivalent to nearly 100%. (**E**) T-sne plot of the RNA-Sequencing data from 99 *Tg[cd4-1:mCherry]* cells that passed the quality control. The points are coloured based on the cluster the cells belong to. Clusters are labelled - 1, 2 and 3. (**F**) Distribution of FSC (top) and SSC (bottom) values in the different clusters. The lines are coloured based on the cluster the cells belong to. In particular, cells in cluster 2 (coloured in green) have higher FSC and SSC values than cells from other clusters. (**G**) Expression of *spi1a*, *spi1b* (*pu.1*), *lck* and *cd4-1* genes plotted on t-sne plot to allow visualisation of expression levels in different cells and clusters. The cell positions are the same as in (**E**). The expression level scale for each gene in log10 count per million (cpm) plus 1 and is indicated at the right of each panel.

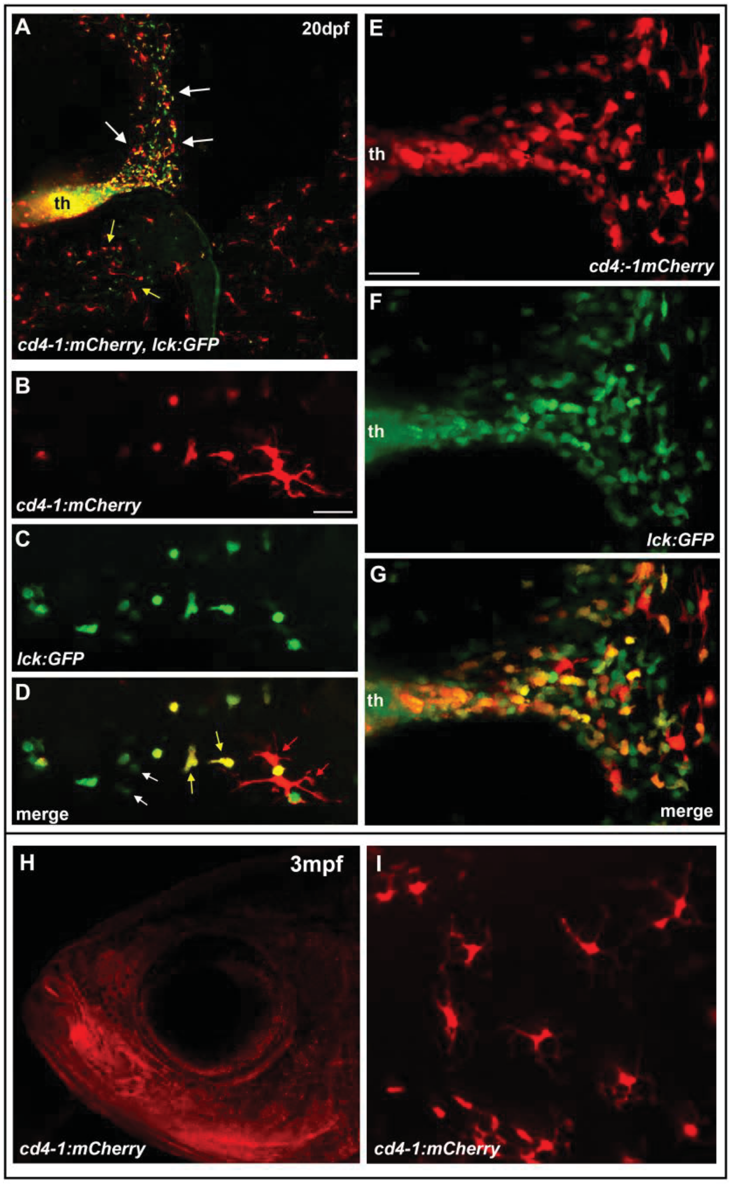

Supplemental Fig. 2. The developmental expression of *Tg(cd4:mCherry)* at 20dpf. (**A**) Composite image of *Tg(cd4-1:mCherry)* and *lck:GFP*. T cells can be seen migrating out of thymus in large numbers (white arrows). T cells and MNPs are also present in the integument (examples indicated by yellow arrows). (**B-D**) High magnification image from skin in the gill cover region showing examples of CD4-1^{+} T cells (yellow arrows), CD4-1^{-} T cells (white arrows) and MNPs (red arrows). (**E-G**) Higher magnification of the corridor of cells migration out of the thymus showing large numbers of T cells in addition to MNPs. (**H**) Extensive network of MNPs is visible throughout adulthood (representative image at 3 months post-fertilisation). (**I**) High magnification image showing the density and morphology of MNPs in the adult integument. th, thymus. Scale bars 20μm.

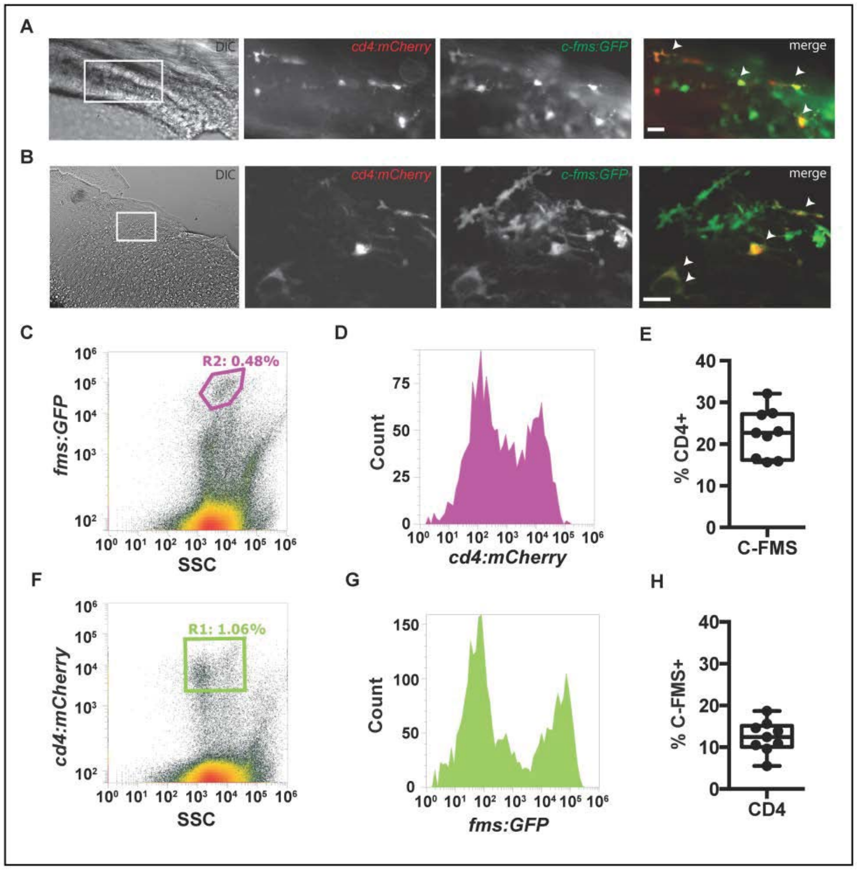

Supplemental Fig. 3. *Tg(cd4-1:mCherry)*$^+$ *fms:GFP*$^+$ MNPs at 20 dpf. (**A**,**B**) Images of *Tg(cd4-1:mCherry)*$^+$ *fms:GFP*$^+$ cells (arrowheads) in the gut (**A**) and skin (**B**). Scale bar 20μm. Left DIC images show boxed area enlarged in fluorescence images. (**C**) Representative *c-fms:GFP*$^+$ population highlighted by region R2. (**D**) Histogram of *Tg(cd4-1:mCherry)* expression in *c-fms:GFP*$^+$ population R2 demonstrating *Tg(cd4-1:mCherry)* expressing sub-population. (**E**) Quantification of percentage of *Tg(cd4-1:mCherry)*$^+$ *c-fms:GFP*$^+$ cells in R2. Percentage calculated by negative gating of *c-fms:GFP*$^+$ single transgenics. Each point is a separate larvae, *n*=8. (**F**) Representative *Tg(cd4-1:mCherry)*$^+$ population highlighted by region R1. (**G**) Histogram of *c-fms:GFP* expression in *Tg(cd4-1:mCherry)*$^+$ population R2 demonstrating *c-fms:GFP* expressing sub-population. (**H**) Quantification of percentage of *c-fms:GFP*$^+$ *Tg(cd4-1:mCherry)*$^+$ cells in R2. Percentage calculated by negative gating of *Tg(cd4-1:mCherry)*$^+$ single transgenics. Each point is a separate larvae, *n*=8.

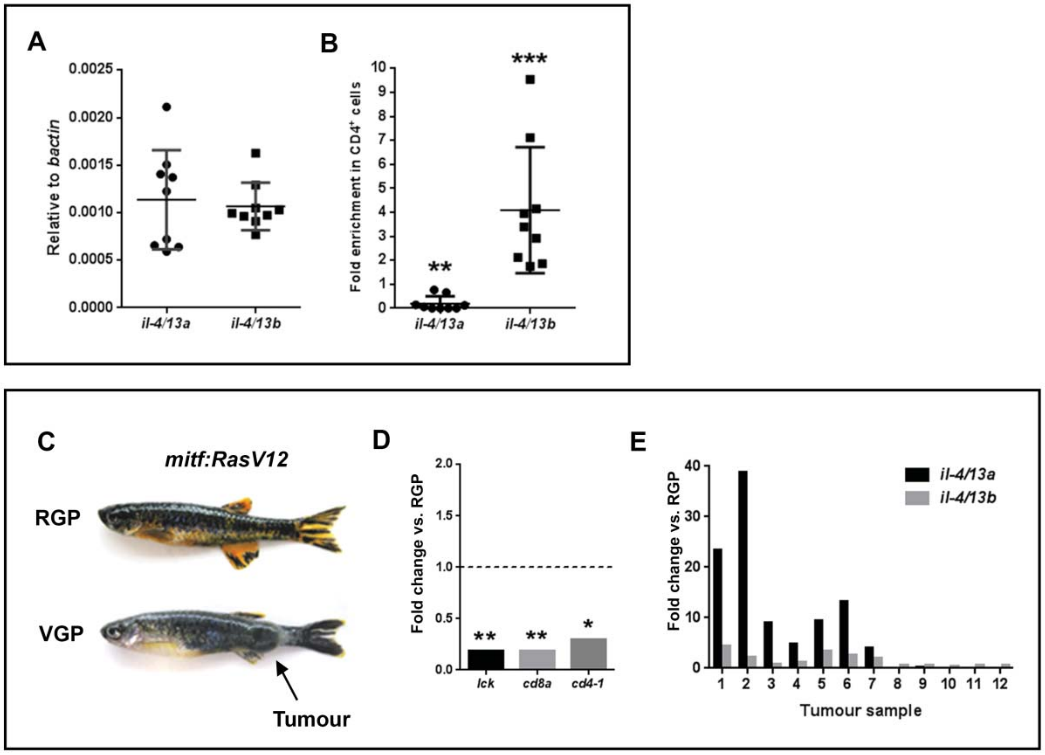

Supplemental Fig. 4. Gene expression in zebrafish gills and melanoma tumours. (**A**) Q-PCR analysis of whole zebrafish gills indicates that *il-4/13a* and *il-4/13b* are constitutively expressed at similar levels. (**B**) The expression of *il-4/13a* and *il-4/13b* in CD4-1$^+$ T cells isolated from the gill. The expression of *il-4/13a* is significantly reduced in CD4-1$^+$ T cells relative to the surrounding gill tissue, while *il-4/13b* expression is significantly enhanced. Error bars equal s.d, n=9, p<0.05. (**C**) The previously established zebrafish model of RAS^{V12} driven melanoma generates radial growth phase (RGP) lesions and occasionally vertical growth phase (VGP) nodular tumours. (**D**) Q-PCR analysis showing that the expression of *lck*, *cd8a* and *cd4-1* is significantly lower in tumours relative to RGP. (**E**) A subset of zebrafish tumours (tumours 1-7) express elevated levels of *il-4/13a*, and often co-express *il-4/13b*. n=12, p<0.05.

Supplementary Video Legends

Video S1. Extended synapsing between T cells and MNPs. Video shows extended membrane contact between T cells (green) and MNPs (red) in the perithymic region. MNPs also become connected to each other temporarily by a cytoplasmic extension. Video length 50 minutes at 0.5 frames/ minute.

Video S2. Detention of T cells by tether like cytoplasmic extensions from MNPs. Video shows a perithymic MNP (red cell) contacting a T cell (green cell) via a long cytoplasmic extension. Video length 52 minutes at 0.5 frames/ minute.
